# Enhanced Extracellular Glutamate and Dopamine in the Ventral Pallidum of Alcohol-Preferring AA and Alcohol-Avoiding ANA Rats after Morphine

**DOI:** 10.3389/fpsyt.2015.00001

**Published:** 2015-01-20

**Authors:** Heidi Kemppainen, Harri Nurmi, Noora Raivio, Kalervo Kiianmaa

**Affiliations:** ^1^Department of Alcohol, Drugs and Addiction, National Institute for Health and Welfare, Helsinki, Finland

**Keywords:** ventral pallidum, morphine, ethanol, GABA, glutamate, dopamine, microdialysis

## Abstract

The purpose of the present study was to investigate the role of ventral pallidal opioidergic mechanisms in the control of ethanol intake by studying the effects of acute administration of morphine on the levels of GABA, glutamate, and dopamine in the ventral pallidum. The study was conducted using the alcohol-preferring Alko Alcohol (AA) and alcohol-avoiding Alko Non-Alcohol (ANA) rat lines that have well-documented differences in their voluntary ethanol intake and brain opioidergic systems. Therefore, examination of neurobiological differences between the lines is supposed to help to identify the neuronal mechanisms underlying ethanol intake, since selection pressure is assumed gradually to lead to enrichment of alleles promoting high or low ethanol intake, respectively. The effects of an acute dose of morphine (1 or 10 mg/kg s.c.) on the extracellular levels of GABA and glutamate in the ventral pallidum were monitored with *in vivo* microdialysis. The concentrations of GABA and glutamate in the dialyzates were determined with a high performance liquid chromatography system using fluorescent detection, while electrochemical detection was used for dopamine. The levels of glutamate in the rats injected with morphine 1 mg/kg were significantly above the levels found in the controls and in the rats receiving morphine 10 mg/kg. Morphine 10 mg/kg also increased the levels of dopamine. Morphine could not, however, modify the levels of GABA. The rat lines did not differ in any of the effects of morphine. The data suggest that the glutamatergic and dopaminergic systems in the ventral pallidum may mediate some effects of morphine. Since there were no differences between the AA and ANA lines, the basic hypothesis underlying the use of the genetic animal model suggests that the effects of morphine detected probably do not underlie the different intake of ethanol by the lines and contribute to the control of ethanol intake in these animals.

## Introduction

It has been hypothesized that mesolimbic dopamine neurons are a neural substrate for the reinforcement produced by ethanol and other substances of abuse (1, 2). Reinforcement from drugs may also be mediated by a downstream pathway of the nucleus accumbens. The mesolimbic dopamine neurons synapse on GABAergic medium spiny neurons of the nucleus accumbens. One population of the medium spiny neurons also expressing enkephalin projects to the ventral pallidum, and consequently the ventral pallidum has been viewed as a final common path for drug reward and as an essential convergent point for hedonic and motivational signaling in the brain (3–5). It seems that depression of the output of medium spiny neurons and activation of the ventral pallidum is common to various drugs of abuse and could be important for their reinforcing properties (4).

In accordance with this view, ethanol, amphetamine, cocaine, and heroin have been shown to suppress extracellular levels of GABA in the ventral pallidum (6–10), and DAMGO, a μ-opioid receptor agonist, has been found to inhibit ventral pallidal GABA release (11). Furthermore, we have recently demonstrated that ventral pallidal μ-opioidergic and GABAergic receptors participate in the regulation of ethanol self-administration (12, 13). In these studies, stimulation of μ-opioid receptors in the ventral pallidum decreased ethanol intake, while blocking of μ-receptors increased ethanol intake. The GABA_A_ receptor agonist muscimol decreased ethanol intake, while administration of the GABA_A_ receptor antagonist bicuculline had the opposite effect. In other studies, ethanol-maintained responding has been found to be reduced by infusions of a ligand binding at GABA_A1_ receptors into the ventral pallidum (14). Moreover, excitotoxic lesions of the ventral pallidum produce changes in the intravenous self-administration of heroin and cocaine (15, 16), and cocaine injected into the ventral pallidum increases locomotor activity (17).

Besides the medium spiny neurons from the nucleus accumbens, other sources of input to the ventral pallidum are glutamatergic projections from the prefrontal cortex as well as mesopallidal dopaminergic neurons from the ventral tegmental area (18–21). Both the glutamatergic and dopaminergic neurons innervating the ventral pallidum have shown some sensitivity to the effects of drugs of abuse, such as heroin, cocaine, and ethanol, suggesting that some effects of these drugs may be mediated by glutamatergic and dopaminergic mechanisms in the ventral pallidum (7, 22, 23).

The purpose of the present study was to clarify further the role of ventral pallidal opioidergic mechanisms in the control of ethanol intake and in the effects of opioids. This was done by studying the effects of acute administration of morphine on the extracellular levels GABA, glutamate, and dopamine in the ventral pallidum using *in vivo* microdialysis. The studies were conducted using selective bred alcohol-preferring Alko Alcohol (AA) and alcohol-avoiding Alko Non-Alcohol (ANA) lines of rats, which represent two non-overlapping phenotypic distributions of voluntary ethanol consumption intake (24). Selectively bred rodent lines differing in ethanol-related phenotypes have been widely used to identify the neuronal mechanisms underlying ethanol abuse (25, 26). Consequently, much effort has been put into the characterization of the AA and ANA rat lines in the search for the neurochemical correlates of the different levels of ethanol intake between the two lines (27). The studies are guided by the basic hypothesis that the selected lines should theoretically differ from each other only in traits that are related to the selected trait. Hence, determination of the neurochemical correlates of the different levels of ethanol intake of the two lines will help to identify the neuronal mechanisms underlying ethanol abuse (27). Earlier studies have revealed strain-specific differences in the opioidergic systems as well as in the neurochemical and behavioral effects of opioids and opioidergic drugs between the AA and ANA rat lines, suggesting a role for the opioidergic systems in their different levels of ethanol intake (27, 28). The present study further pursued the opioidergic involvement focusing on the opioidergic systems in the ventral pallidum of the two rat lines.

## Materials and Methods

### Animals

Male alcohol-preferring AA and alcohol-avoiding ANA rats (National Institute for Health and Welfare, Helsinki, Finland) from generations F_94–95_ were used in the present study. The rats were from 2 to 4 months of age and weighed from 245 to 392 g at the time of surgery. The rats were housed in groups of four or five in plastic cages (Makrolon IV 56 cm × 34 cm × 19 cm) until the implantation of the microdialysis guide cannula. After the surgery, each rat was caged individually in a Plexiglas cage (35 cm × 23 cm × 30 cm) until the end of the experiment with food [SDS RM1 (E) SQC, Witham, Essex, England] and water available *ad libitum*. Ambient temperature was kept at 22 ± 2°C and humidity at 55 ± 10%. The light/dark cycle was 12/12 h (lights on at 06:00 hours). The experiments were conducted during the light phase of the cycle. All experimental procedures using animals were carried out in accordance with the European Communities Council Directive (2007/526/EEC) and the National Animal Welfare Act and were reviewed and approved according to the Act on the Use of Animals for Experimental Purposes (62/2006) in the National Animal Ethics Committee in Finland.

### Morphine treatment

The rats received morphine acutely 1 or 10 mg/kg s.c. in a volume of 1 ml/kg in saline. The controls were given an equal volume of saline.

### Surgery

The rats were anesthetized with halothane (4% during induction for 4 min and 1.5–2% during the surgery) and attached to a stereotactic frame for implantation of a guide cannula (CMA/12, CMA Microdialysis, Stockholm, Sweden) into the brain just above the ventral pallidum. Prior to the surgery, lidocaine was applied to the surgical site (s.c.). The coordinates for the microdialysis probe, as given in the atlas of the rat brain by Paxinos and Watson (29), were 0.8 mm posterior to bregma, 3.2 mm lateral to midline, and 5.1 mm below the dura. They were selected on the basis of the neurochemical work on the ventral pallidum and hedonic reward (30). The guide cannula was fastened to the skull with two stainless steel screws and dental cement. Body temperature was kept constant at 37°C during the procedure with a thermostatically controlled thermal mattress. The rats were administered carprofen (Rimadyl^®^, 0.1 mg/kg s.c.) before and the day after the surgery. After the surgery, the rats were held in individual cages. They were allowed to recover for at least 5 days during which they were habituated to the experimental procedures by tethering them to a counterbalancing arm several times.

### *In vivo* microdialysis

Microdialysis was performed in the home cages. A probe (CMA/12, membrane length 2 mm, o.d. 0.5 mm, polycarbonate membrane with a 20 kDa cut-off, CMA Microdialysis, Stockholm, Sweden) was inserted into the guide cannula at 1600 h on the day preceding the experiment and left there without perfusion overnight. In the morning, the rats were tethered to the counterbalancing arm and a sterilized and modified Ringer solution (148 mM NaCl, 2.7 mM KCl, 1.2 mM CaCl_2_, 0.85 mM MgCl_2_ ⋅ 6H_2_O, pH ~7.00) was perfused through the probe with a flow rate of 1.5 μl/min using a CMA 100 microinjection pump. Samples were collected with a refrigerated sample collector (Univentor 820, Zejtun, Malta) every 20 min. Baseline samples were collected for 2 h; thereafter the rats were given an injection of morphine, 1 or 10 mg/kg, or saline, and samples were collected for the next 4 h. The samples were then divided into three vials for separate analysis of GABA, glutamate, and dopamine, and stored at −70°C until analysis.

The neuronal origin of GABA was evaluated with high potassium chloride solution (31). After finishing an experiment, 60 mM KCl (in Ringer) solution was perfused through the probe and samples were collected for another 2 h. The concentration of GABA was measured in eight samples.

### Analyses of GABA, glutamate, and dopamine

The concentrations of GABA, glutamate, and dopamine in the microdialysis samples were determined with high performance liquid chromatography (HPLC). The HPLC system consisted of an isocratic pump with a degasser unit and a refrigerated autoinjector (Hewlett Packard 1100 series, Palo Alto, CA, USA).

The concentrations of GABA and glutamate were determined in separate runs using fluorescent detectors (Waters 2475 Multi λ, Waters, Milford, MA, USA for GABA, and CMA/280, CMA Microdialysis, Stockholm, Sweden for glutamate) equipped with an 8 μl flow-cell and was operated at an excitation wavelength of 330 nm and emission at 440 nm. The columns were Discovery^®^RP Amide C16 150 × 3 mm i.d., a particle size of 5 μm (Supelco, Bellefonte, PA, USA) for GABA, and Hichrom Hypersil H5ODS-200M 200 × 1 mm i.d., a particle size of 5 μm (Hichrom, Berkshire, UK) for glutamate. The microdialysis samples were mixed with 2 μl of o-phthalaldehyde-β-mercaptoethanol for pre-column derivatization, and the injection volumes were 4.5 and 14 μl for glutamate and GABA, respectively. The mobile phase for glutamate was 0.3 M acetic acid buffer containing 16% (v/v) acetonitrile and 0.1 mM Na-ethylenediaminetetraacetic acid (EDTA) at pH 5.85, and for GABA, 0.1 M acetic acid buffer containing 45% (v/v) methanol and 0.1 mM Na-EDTA at pH 5.12. The flow rate of the mobile phase was set to 0.07 and 0.3 ml/min for glutamate and GABA, respectively. The chromatograms were acquired and processed with Class VP software v 6.12 (Shimadzu Corporation, Kyoto, Japan).

Concentrations of dopamine were measured with amperometric detector (Antec Intro, Antec BV, Leyden, The Netherlands) with a glassy-carbon VT-03 cell. The glassy-carbon working electrode was set to a +700 mV vs. Ag/AgCl reference electrode. The column used was MIC10-3-C18, 100 mm × 1 mm i.d., with a particle size of 3 mm (Hypersil, LC-Packings, The Netherlands). The mobile phase was 11% (v/v) methanol in 50 mM phosphate/50 mM citric acid buffer, 0.15 mM EDTA, 10 mM NaCl, and 0.22 mM sodium octylsulphonate at pH 4.8. The solution was filtered through a polyvinylidene fluoride filter with 0.45-mm pores (Millipore, Milford, MA, USA). The flow rate of the HPLC pump was set to 35 μl/min, and the injection volume was 15 μl. The limit of detection for dopamine was 0.35 fmol/ml. The chromatograms were processed with Waters 820 Maxima Software, v 3.31 (Waters, Milford, MA, USA).

The microdialysis data (micrometer or nanometer) were converted into percentages of the baseline consisting of the mean of the four baseline samples, and not corrected for probe recovery.

### Histology

The positions of the probes were verified by fixing the brain in 10% formalin and making frozen 100 μm coronal sections stained with thionine after completion of the experiments. Prior to the experiment, it was decided that data would be analyzed only from the rats in which at least 50% of the probe membrane was verified to be in the ventral pallidum.

### Chemicals and reagents

Morphine-HCl was purchased from University Pharmacy (Helsinki, Finland), 2-bromo-2-chloro-1.1.1trifluoroethane (Halothane BP) from Nicholas Piramal India Ltd. (Chennai, India), karprofen (Rimadyl Vet) from Vericore Ltd. (Dundee, England), and Lidocaine from Orion Pharma (Espoo, Finland). o-Phthalaldehyde was obtained from Pickering Laboratories (CA, USA) and 2-mercaptoethanol from Merck KGaA (Darmstadt, Germany). The other common reagents were of HPLC quality and were obtained from Sigma-Aldrich (St. Louis, MO, USA) or Merck (Darmstadt, Germany).

### Statistical analysis

The microdialysis data were analyzed with a mixed-design three-way analysis of variance (ANOVA) with treatment (saline and morphine) and rat line (AA and ANA) as the between-subjects factors, and measuring interval (time) as the within-subjects repeated measure. After finding significant main effects, differences within the rat lines were examined with a subsequent repeated measures two-way ANOVA. *Post hoc* comparisons between the group means were conducted using Tukey’s test when appropriate. The level of significance was set at 0.05.

## Results

**Figure 1:**
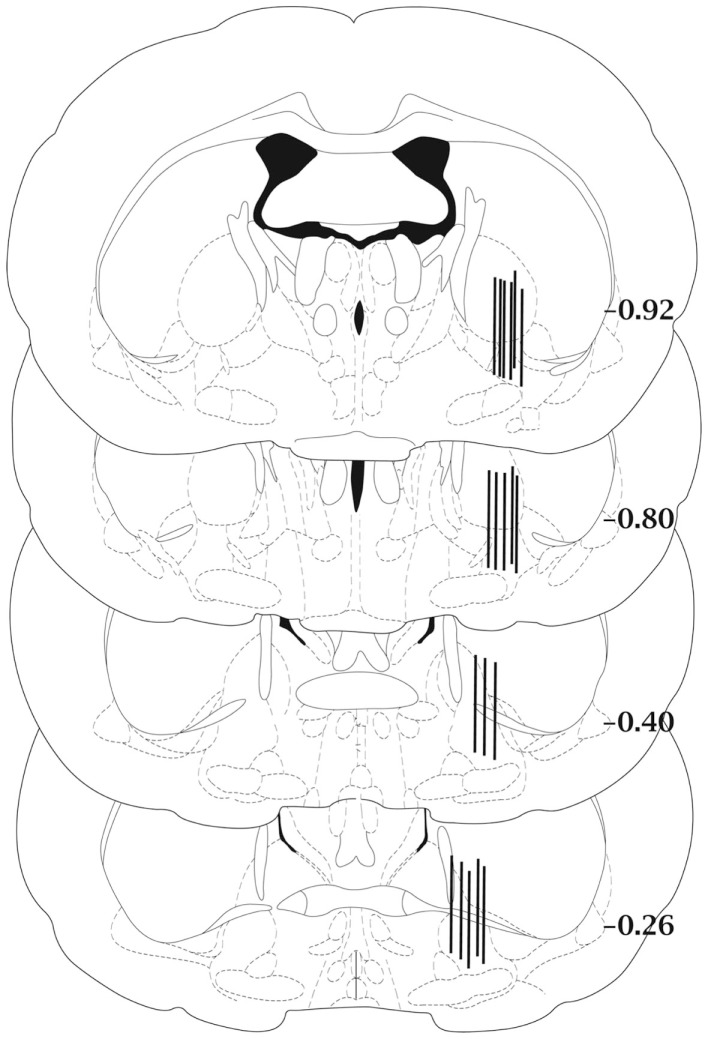
**Schematic diagram of representative microdialysis probe placements in the ventral pallidum**. Not all probe placements are shown. Each bar represents two individual rats and is fitted to the coronal section closest to the actual anterior–posterior placement of the probe. Anterior–posterior placement of the sections is indicated relative to bregma. Coronal sections were adapted from the atlas of Paxinos and Watson (29).

Histological examination of the microdialysis probe placements showed that all samples had been collected from the central or caudal portions of the ventral pallidum (Figure 1).

**Figure 2:**
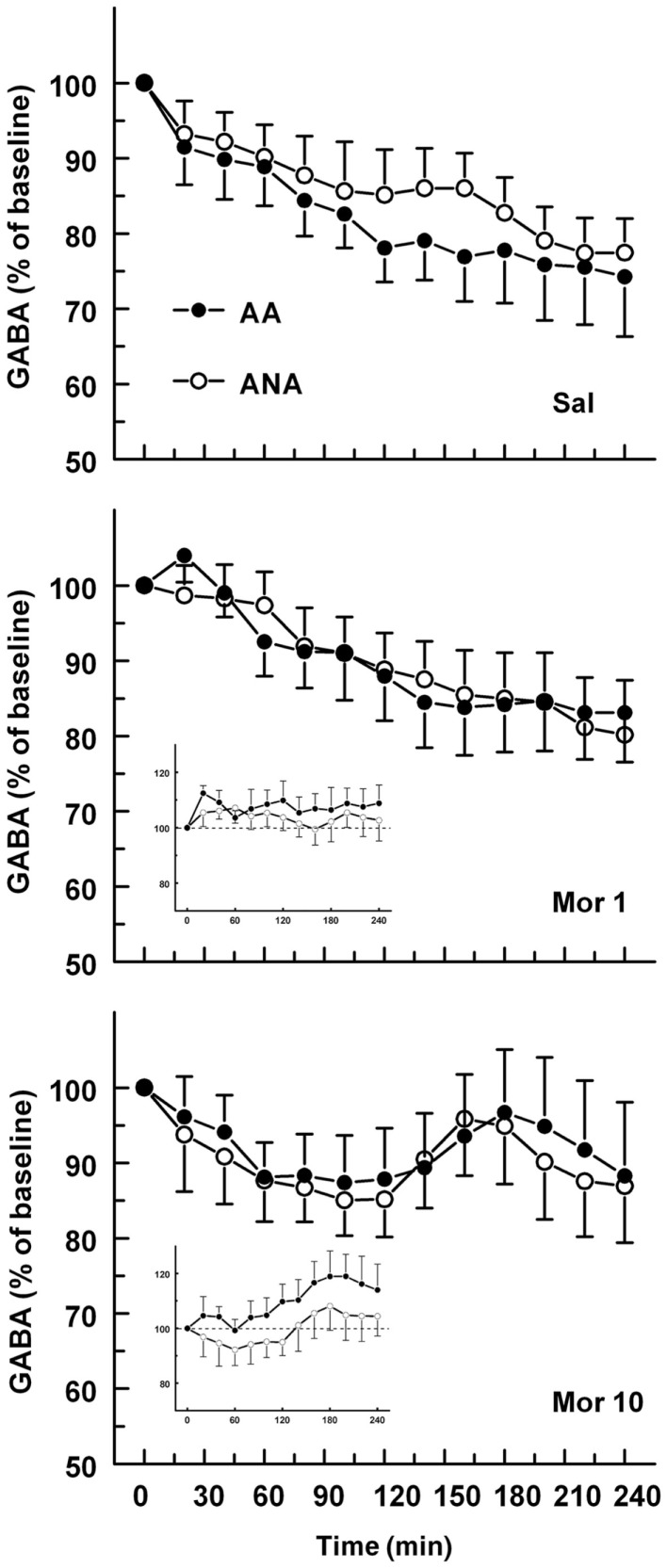
**The effect of an acute dose of morphine (1 or 10 mg/kg s.c.; groups Mor 1 and Mor 10) on the extracellular concentrations of GABA in the ventral pallidum of alcohol-preferring AA and alcohol-avoiding ANA rats**. The controls (Sal) received an equal volume of saline. The inset shows the data as a difference from the controls. The values are expressed as a percentage of the baseline consisting of the mean of the last four baseline samples. The moving average of three time points ± SEM is given. Number of animals in each group: AA Sal = 8, Mor1 = 7, Mor10 = 8; ANA Sal = 7, Mor1 = 7, and Mor10 = 8.

The basal concentrations of GABA in the ventral pallidum were 143.86 ± 36.30 and 102.60 ± 20.60 nM for the ANA and AA lines, respectively. The basal GABA levels did not differ significantly. Figure 2 shows the effect of an acute dose of morphine on the extracellular concentration of GABA in the ventral pallidum of AA and ANA rats. Administration of morphine did not modify the levels of GABA in either AA or ANA rats. Application of 60 mM KCl doubled the concentration of GABA relative to the baseline, verifying that the bulk of the measured concentrations of GABA were of neuronal origin and hence the concentrations reflected true changes in neuronal activity.

**Figure 3:**
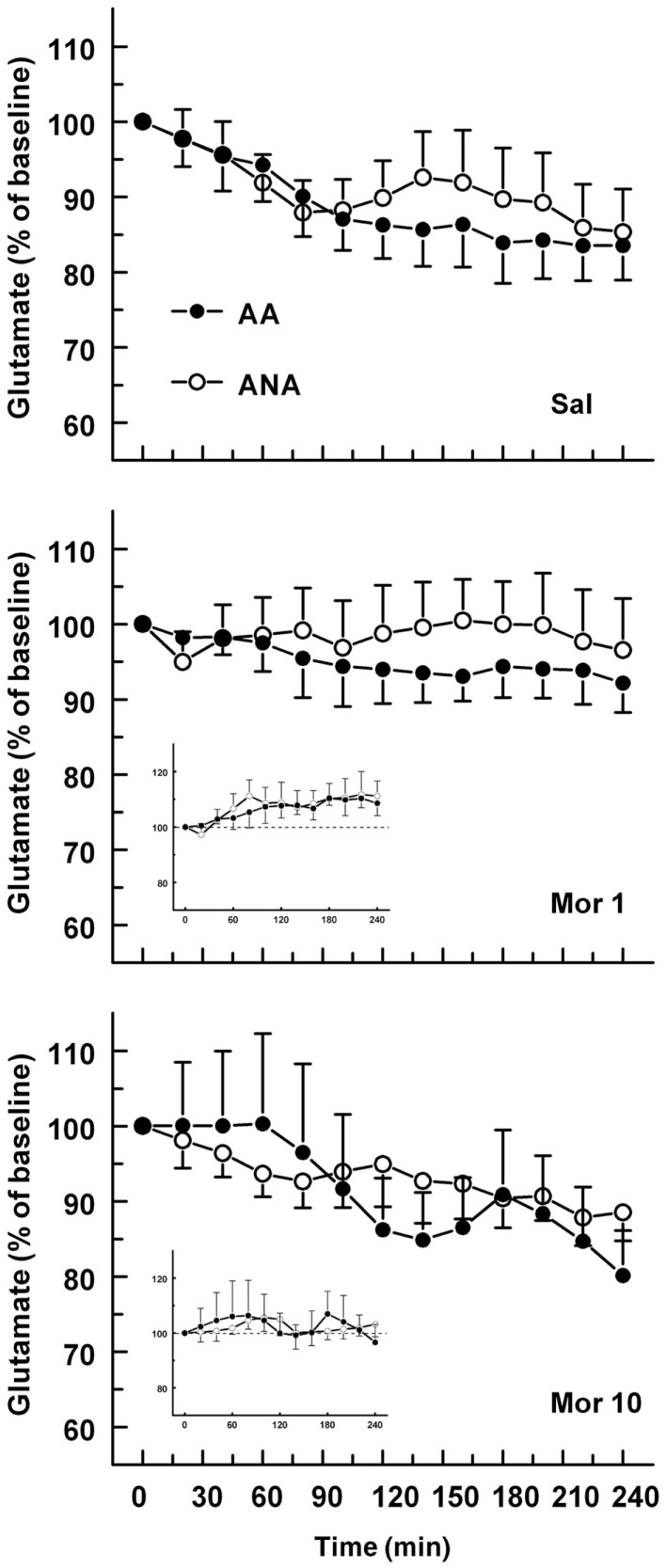
**The effect of an acute dose of morphine (1 or 10 mg/kg s.c.; groups Mor 1 and Mor 10) on the extracellular concentrations of glutamate in the ventral pallidum of alcohol-preferring AA and alcohol-avoiding ANA rats**. The controls (Sal) received an equal volume of saline. The inset shows the data as a difference from the controls. The values are expressed as a percentage of the baseline consisting of the mean of the last four baseline samples. The moving average of three time points ± SEM is given. Number of animals in each group: AA Sal = 8, Mor1 = 7, Mor10 = 9, ANA Sal = 7, Mor1 = 7, and Mor10 = 6.

The basal concentrations of glutamate were 3.48 ± 0.50 and 4.93 ± 0.80 μM for the ANA and AA lines, respectively. The basal glutamate levels did not differ significantly. The effect of an acute dose of morphine on the extracellular concentration of glutamate in the ventral pallidum of AA and ANA rats is shown in Figure 3. Morphine modified the extracellular concentration of glutamate significantly [*F*(2,52) = 4.496, *p* = 0.016, for treatment] in the rats but not in either line when tested separately. The levels of extracellular glutamate in the rats injected with morphine 1 mg/kg were significantly (*p* < 0.05, Tukey’s test) above the levels of the controls and the rats receiving morphine 10 mg/kg.

**Figure 4:**
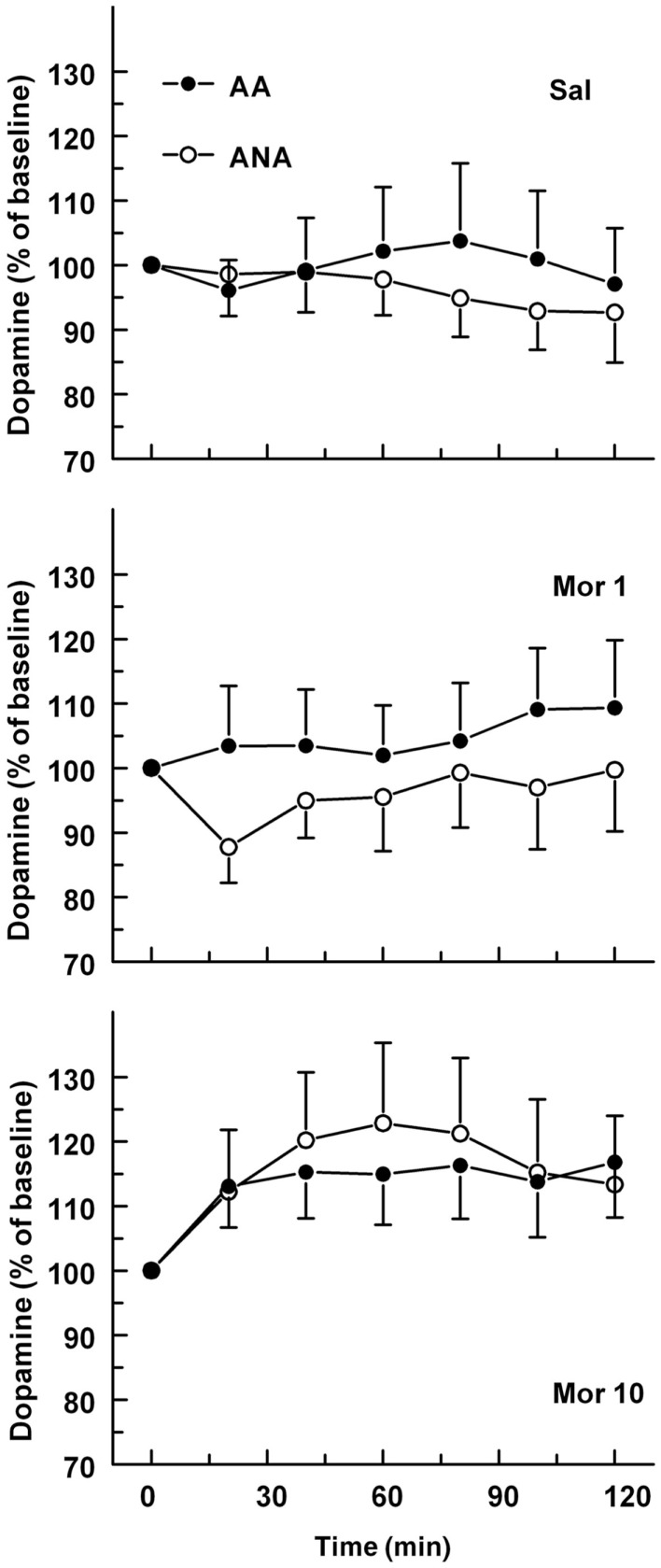
**The effect of an acute dose of morphine (1 or 10 mg/kg s.c.; groups Mor 1 and Mor 10) on the extracellular concentrations of dopamine in the ventral pallidum of alcohol-preferring AA and alcohol-avoiding ANA rats**. The controls (Sal) received an equal volume of saline. The values are expressed as a percentage of the baseline consisting of the mean of the last four baseline samples. The moving average of three time points ± SEM is given. Number of animals in each group: AA Sal = 7, Mor1 = 10, Mor10 = 14, ANA Sal = 11, Mor1 = 7, and Mor10 = 9.

The basal concentrations of dopamine in the ventral pallidum were 1.40 ± 0.30 and 1.49 ± 0.40 nM for the ANA and AA lines, respectively. The basal dopamine levels did not differ significantly. The effect of an acute dose of morphine on the extracellular concentration of dopamine in the ventral pallidum of AA and ANA rats is shown in Figure 4. Morphine increased extracellular levels of dopamine [*F*(2,52) = 4.67, *p* = 0.014, for treatment]. Analyzing the lines separately revealed that morphine increased the level of dopamine significantly in the ANA line [*F*(2,24) = 4.629, *p* = 0.02, for treatment]. Maximal increase was seen 60 min after administration of the morphine. The concentrations of dopamine in the ANA rats receiving morphine 10 mg/kg were significantly higher than those in the controls (*p* < 0.05, Tukey’s test).

## Discussion

The objective of the present investigation was to use the model of selective bred alcohol-preferring AA and alcohol-avoiding ANA lines of rats to clarify the role of ventral pallidal opioidergic mechanisms in the control of ethanol intake and in the effects of opioids by studying the effects of acute administration of morphine on the extracellular levels of GABA, glutamate, and dopamine in the ventral pallidum. In these studies, ventral pallidal levels of glutamate and dopamine were significantly elevated after morphine. Morphine did not, however, modify the levels of GABA. The rat lines did not differ significantly in the effects of morphine. The rats receiving 1 mg/kg of morphine showed higher levels of glutamate than the other groups.

Drug-induced increase in dopamine release in the nucleus accumbens is believed to be a critical condition to produce reinforcement (1). Numerous studies, however, suggest that the reinforcing effects of ethanol are probably not mediated exclusively through the dopaminergic neurons. For instance, destruction of the dopaminergic terminals in the nucleus accumbens does not attenuate acquisition or maintenance of ethanol self-administration behavior (32, 33). Mechanisms downstream of the nucleus accumbens may also participate in the mediation of reinforcement from ethanol. The mesolimbic dopamine neurons synapse on the GABAergic medium spiny neurons in the nucleus accumbens (3–5). One population of the medium spiny neurons also expressing enkephalin projects to the ventral pallidum. According to the prevailing hypothesis, dopamine released in the nucleus accumbens inhibits GABAergic medium spiny neurons projecting to the ventral pallidum and the resulting suppression of GABA release is common to various drugs of abuse and could be important for their reinforcing properties (4). In accord with this hypothesis, opiates morphine, and heroin, as well as other drugs of abuse such as amphetamine, cocaine, and ethanol have been shown to suppress extracellular levels of GABA or inhibit GABAergic eISPCs in the ventral pallidum (6–11, 34). In theory, morphine can induce a reduction in the ventral pallidal levels of GABA by increasing the release of dopamine in the nucleus accumbens, thereby increasing inhibitory drive on medium spiny neurons (35), or by stimulating μ-opioid receptors on medium spiny neurons in the nucleus accumbens or presynatically in the ventral pallidum (3, 10, 36). Furthermore, dopaminergic input from the mesencephalon as well as glutamatergic terminals from the prefrontal cortex possibly also exert their effects on GABAergic tone in the ventral pallidum (18, 20, 21).

In the present study, the extracellular levels of GABA in the ventral pallidum were, however, not modified significantly by morphine in either rat line. There was only a slight trend for increased levels of GABA during the latter half of the 240 min sampling time after 10 mg/kg of morphine. Therefore, the present study is not consistent with earlier studies, where morphine (3 mg/kg) or self-administered heroin suppressed extracellular levels of GABA significantly (7, 34), or where DAMGO inhibited GABA release in the ventral pallidum (11). The finding that morphine did not modify the ventral pallidal levels of GABA is in contrast to the hypothesis that the reinforcement from drugs of abuse comes from suppression of GABA release in the ventral pallidum. The present data are not in line with our earlier behavioral data either. We have recently demonstrated that ventral pallidal μ-opioidergic and GABAergic receptors participate in the regulation of ethanol self-administration (12, 13). In these studies, stimulation of μ-opioid receptors in the ventral pallidum decreased ethanol intake, while blocking μ-receptors increased ethanol intake. The GABAA receptor agonist muscimol decreased ethanol intake, while administration of the GABAA receptor antagonist bicuculline had the opposite effect. The discrepancy between the studies and the reason for the lack of effect from morphine in the present study remain unclear. It is possible that subtle changes in the extracellular levels of GABA were not detected by the method used in the present study. This is, however, unlikely, since in another study by the present authors, ethanol was found to lower the ventral pallidal levels of GABA in ANA rats (8). Since both in the present study and in the study by Caillé and Parsons (7) morphine was administered subcutaneously, the route of morphine administration probably is not a confusing factor in the studies.

Glutamatergic afferents from the prefrontal cortex to dopaminergic neurons provide input to the reward circuitry, and glutamatergic projection from the prefrontal cortex to the ventral pallidum has been described as well (21, 37). The effects of morphine or other drugs of abuse on the overflow of glutamate in the ventral pallidum are not, however, well characterized. In the present study, the rats receiving morphine 1 mg/kg showed significantly higher levels of glutamate in the ventral pallidum than what was seen in the controls and in the rats receiving morphine 10 mg/kg, suggesting enhancement of extracellular glutamate after the lower dose. There was no difference in the effect between the two lines. We have earlier reported a morphine-induced increase in the extracellular levels of glutamate in the ventral tegmental area, which demonstrates the interaction of the opioidergic and glutamatergic systems also at the level of ventral tegmental area (38). The enhancement of ventral pallidal glutamate found here seems to be concurrent with morphine-induced (1 mg/kg) locomotor activity (39). Activation of glutamatergic receptors in the ventral pallidum has earlier been shown to be involved in the hypermotility response to central nervous system stimulants (40). Somewhat in line with the present findings, self-administered heroin did not initially have any effect on the levels of glutamate in the ventral pallidum in an earlier study, but an increase was detected subsequent to 1 h of self-administration (7). It was speculated the delayed onset may signify either a long-loop feedback mechanism or a desensitization of mechanisms regulating glutamatergic input. Sizemore and others reported small increases in glutamate levels during cocaine self-administration sessions (22). Ethanol did not, however, modify significantly the ventral pallidal levels of glutamate (8). Furthermore, in some studies, pallidal microinjections of pharmacological agents selective to metabotropic glutamate receptor 7 have been found to modulate the rewarding effects of cocaine (9). Nevertheless, these findings give support to the involvement of ventral pallidal glutamatergic mechanisms in the effects and self-administration of drugs of abuse, but this needs further characterization.

Dopaminergic innervation of the ventral pallidum arises predominantly from the ventral tegmental area (20, 41). Morphine increased the extracellular levels of dopamine in the ventral pallidum in the present study. Although the effect of morphine on dopamine reached significance in the ANA but not in the AA line, the conclusion of a different sensitivity of the lines to the effect of morphine on ventral pallidal dopamine is not justified. Consequently, the interaction of opioidergic and dopaminergic mechanisms on the level of ventral pallidum probably does not play a role in the different levels of intake of ethanol by the rat lines. Morphine has earlier been reported to increase the release of dopamine in the nucleus accumbens of AA and ANA rats, suggesting a role for the mesolimbic dopamine neurons in the reinforcing effects of morphine (35, 42). Moreover, a morphine-induced increase in accumbal dopamine was more prominent than in the ventral pallidum. The two lines did not, however, differ in the effect of an acute dose of morphine on accumbal dopamine (35). In studies by others, intraperitoneal administration of ethanol, as well as systemic and intra-ventral pallidal administration of cocaine has been shown to increase the extracellular levels of dopamine in the ventral pallidum, suggesting the involvement of mesopallidal dopaminergic neurons in the actions of ethanol and cocaine (43, 44). According to the present findings, some effects of morphine may also be mediated by the mesopallidal dopaminergic neurons.

Microdialysis has been used widely to sample various neurotransmitter substances from the brain. It has, however, been pointed out that there may be problems in detecting subtle changes in amino acid levels with the *in vivo* microdialysis technique, possibly because a portion of amino acids in the extracellular space is derived from sources that are not directly involved in neurotransmission (45). This does not necessarily argue against the functional significance of changes in extracellular amino acid levels in the brain (46). According to Bourdelais and Kalivas (47), basal extracellular levels of GABA in the ventral pallidum may be maintained, at least partially, by ongoing neuronal activity, and that at least 50% of extracellular GABA of the ventral pallidum results from neurotransmission. In accordance, this and our previous study showed that 60 mM KCl doubled the concentration of GABA in the ventral pallidum, and hence the alterations in the concentrations of GABA reflected true changes in neuronal activity (8, 31). Our preliminary findings based on the use of a low calcium Ringer solution or local infusion of 100 μM baclofen support a similar conclusion.

Dialysis sites in the present study were located in the central and posterior parts of the ventral pallidum. These areas were found to be responsive for μ-opioid receptor agonists and an antagonist in a previous study by the present authors (8). Earlier brain mapping work has suggested functional differences within the ventral pallidum in the mediation of reward (3, 30). According to these studies posterior ventral pallidum μ-opioid neurotransmission in particular encodes hedonic reward and motivation.

Taken together, in the present study based on the use of the genetic animal model of alcohol-preferring AA and alcohol-avoiding ANA rats to clarify the role opioidergic mechanism in ethanol intake, acute administration of morphine was associated with increased levels of glutamate and dopamine in the ventral pallidum, while no changes in the levels of GABA were observed. The higher pallidal concentrations of glutamate after morphine are in line with earlier studies. The functional significance of the detected changes in the levels of glutamate may, however, be challenged due to the potential problems arising from monitoring glutamate with microdialysis (45). In contrast, morphine-induced increase in the levels of dopamine along with similar findings by others after administration of ethanol and cocaine suggests that the mesopallidal dopamine neurons may also mediate some effects of these drugs. Since the AA and ANA rat lines did not differ in the effects of morphine, the results do not provide additional information on the understanding of the role of opioidergic mechanisms in the different levels of intake of ethanol by the rat lines or more generally in the control of ethanol intake by rats.

## Conflict of Interest Statement

The authors declare that the research was conducted in the absence of any commercial or financial relationships that could be construed as a potential conflict of interest.
